# Supplementary material for: New Insight into the Gas Phase Reaction Dynamics in Pulsed Laser Deposition of Multi-Elemental Oxides
Source: Materials (Basel). 2022 Jul 12;15(14):4862. doi: 10.3390/ma15144862 (PMC9320868; doi:10.3390/ma15144862)
Supplement: Supplementary file 1 [file materials-15-04862-s001.zip › materials-1696353-supplementary.pdf]

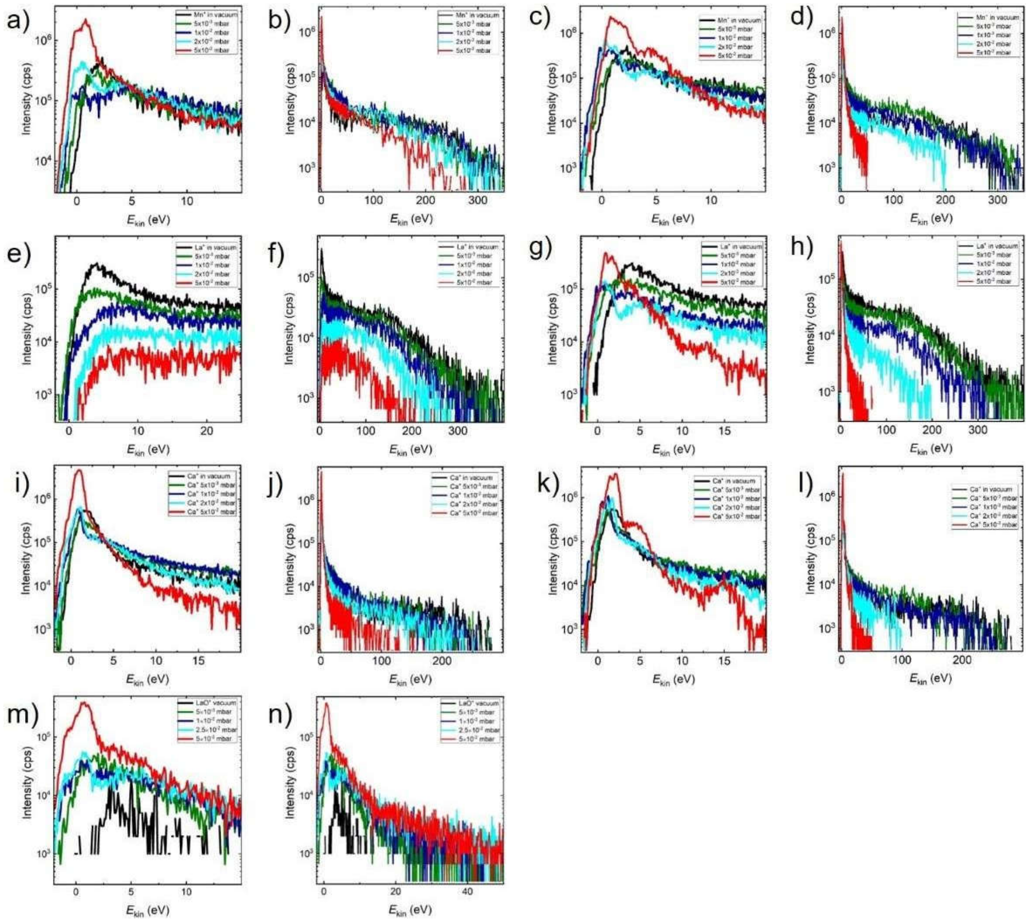

Figure S1. The ion energy distributions (low KE range, full measurements range) for (a, b) Mn^+ in 15 O_2, (c, d) Mn^+ in Ar, (e, f) La^+ in O_2, (g, h) La^+ in Ar, (i, j) Ca^+ in O_2, (k, l) Ca^+ in Ar, and (m, n) LaO^+ in 16 O_2 at all measured deposition pressures.

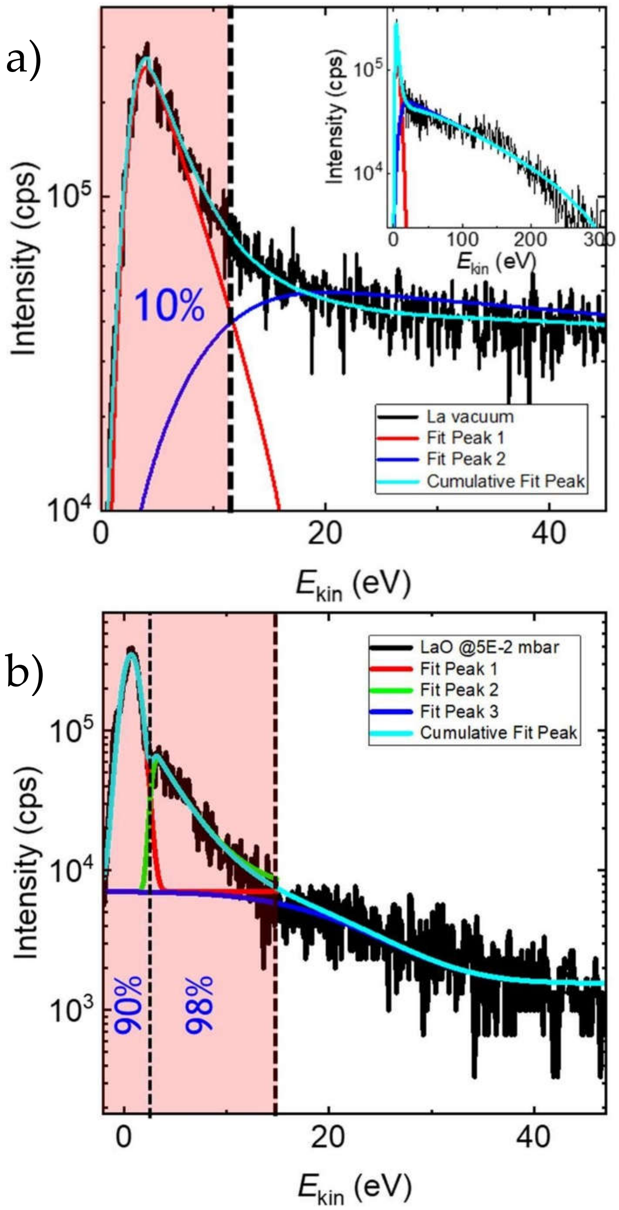

Figure S2. Fitting of ion energy distributions for a) La^+ ablated in vacuum and b) LaO^+ ab- lated at $5x10^{-2}$ mbar O_2 using Maxwell-Boltzmann (MB) distributions. The entire La^+ vac- uum-distribution can be described by two MB velocity distributions, the LaO^+ distribution with three. The fitting with three MB functions suggests an approximately 50eV wide win-dow for chemical activity. Between 0-5 eV more than 90% of the measured LaO^+ species have been formed, and between 5 and 15 eV 98 % indicating that most LaO^+ species have been formed within a kinetic energy window of 15eV. For the La^+ species, the maximum E_{kin} is at ~2.6 eV and the second maximum at ~22 eV. The origin of the slow species is probably related to thermally emitted species after the termination of the laser pulse whereas the faster species are emitted and accelerated within the timescale of the laser pulse and subse-quently slowed down. Within a kinetic energy window of 12 eV, only about 10% of all La^+ species are located.
